# Koori voices: self-harm, suicide attempts, arrests and substance use among Aboriginal and Torres Strait Islander adolescents following residential treatment

**DOI:** 10.1186/s40352-020-0105-x

**Published:** 2020-02-07

**Authors:** S. Nathan, K. Maru, M. Williams, K. Palmer, P. Rawstorne

**Affiliations:** 1 School of Public Health and Community Medicine, UNSW, Sydney, Australia; 2 Graduate School of Health, University of Technology, Sydney, Australia; 3 Ted Noffs Foundation, Sydney, Australia

**Keywords:** Aboriginal and Torres Strait islander, Indigenous, Adolescents, Young people, Drugs, Alcohol, Residential treatment, Suicide, Self-harm, Outcomes

## Abstract

**Background:**

Complex interacting social, economic and historical factors influence the availability and uptake of alcohol and drugs, including among Indigenous communities. Self-harm and suicide as well as homelessness and incarceration, can both precede and result from drug and alcohol use. Rates of self-harm, suicide and incarceration among Aboriginal and Torres Strait Islander people in Australia are among the highest in the world and drug and alcohol treatment programs need to address these underlying complexities. This study examines whether an ‘holistic’ residential drug and alcohol treatment program for adolescents, with over 30% of clients identifying as Aboriginal and Torres Strait Islander, can improve outcomes post-discharge, including reducing self-harm, suicide attempts, arrests and alcohol and drug use. The program addresses substance use, mental health, employment, accommodation, social/community and family life. Program admission and 3 months’ post-discharge data from 2007 to 2016 were analysed.

**Results:**

From 2007 to 2016, 619 Aboriginal and Torres Strait Islander young people were admitted to the program; 247 stayed in the program for 30 days or more; 89 were successfully followed up three months post-discharge to determine whether there was a significant improvement from baseline using the McNemar’s Test and the Wilcoxon Signed Ranks Test. On admission, 18 people (20%) of the study population reported attempting suicide in the last 3 months and 23 people (30%) reported self-harm. Most had been engaged in the criminal justice system, with 67 people (75%) having been to court and 62 people (70%) arrested one or more times in the past 3 months, with 35 people (41%) in unstable housing, reporting having lived in three or more places in the previous 6 months. At 3 months post-treatment, all (*n* = 18) who reported suicide attempts in the 3 months prior to admission reported no attempts in the prior 3 months at follow-up. There was also a significant reduction in self-harm with 23 young people out of the 27 who reported self-harm at baseline not reporting self-harm at follow up (85%) and in the proportion of adolescents who reported using cannabis, amphetamines and alcohol, as well as a reduction in the proportion who reported being arrested.

**Conclusions:**

The findings provide support for an ‘holistic’ residential treatment program as an approach to improve health and related outcomes for Aboriginal and Torres Strait Islander young people. In addition to a focus on multiple aspects of a young person’s life in treatment, culturally relevant modes of treatment and support should be a future focus to further strengthen programs when Aboriginal and Torres Strait Islander young people are over-represented in the client group.

## Background

Complex interacting social, economic and historical factors influence the availability, uptake and use of alcohol and drugs as well as the strategies used to reduce associated risks and harms. Factors, such as homelessness, lower socioeconomic status, incarceration and violence, can both precede and result from drug and alcohol use (Fox, Oliver, & Ellis, 2013; Whitesell, Bachand, Peel, & Brown, 2013). In Australia, a harm minimisation framework underpins many of the approaches to drug and alcohol use (Australian Department of Health, 2011), however, there remains high levels of alcohol consumption in the general community (AIHW, 2014), and high rates of illicit drug use compared to many other countries (AIHW, 2011a).

Within the broader Australian context, the health and wellbeing of Aboriginal and Torres Strait Islander people is worsening with the national government unable to meet its own targets to reduce health and social inequity (CoA, 2017). Aboriginal and Torres Strait Islander peoples constitute 2.8% of the total population (ABS, 2016; Madden, Tickle, Jackson Pulver, & Ring, 2012). For 60,000 years before colonisation in 1788, the 300-plus inter-dependent nation groups flourished with systems for nutrition, law, relationships, identity and survival. After the British proclaimed ownership (Kidd, 1997; Perkins, 2010), introduced infectious diseases and alcohol, restriction from traditional foods, massacres, poisonings and lack of healthcare, resulted in innumerable deaths (Attwood, 2005; Elder, 2003). It was not until 1967 that Aboriginal and Torres Strait Islander people were considered citizens. Despite never ceding sovereignty Aboriginal and Torres Strait Islander people continue to suffer profound injustices in Australian society (Burgess & Johnstone, 2007; Strelein, 2009).

Over-representation in correctional centres (ABS, 2017) and high rates of youth detention (AIHW, 2016) reinforce deep wounds created by deliberate removal of children from families under past policies. These ‘Stolen Generations’ have significantly higher alcohol and drug use, post-traumatic stress disorder and other illnesses with inadequate resources for therapeutic care than the general Australian population (Human Rights and Equal Opportunities Commission, 1997; Phillips, 2007). Emerging evidence now shows a biological as well as social effect of trauma, with trauma effects extending inter-generationally to individuals and their families (Atkinson, 2002; Atkinson, 2009). International research shows higher levels of trauma, mental illness and suicide among Indigenous peoples removed from family and has been associated with poorer health among those in Canada, New Zealand and Australia (Elias et al., 2012; King, Smith, & Gracey, 2009; Waldram, Herring, & Young, 2006). The loss of connection to cultural life, trauma, racism and social exclusion contribute to poorer health including harmful alcohol and drug consumption (Dixson et al., 2018; Waldram, Herring & Young, 2006). The health status of Indigenous populations worldwide are poorer than non-Indigenous populations with similar alcohol and drug use patterns (Pulver et al., 2010). A recent systematic review examined suicide rates among Indigenous peoples in 30 countries and territories (Pollock, Naicker, Loro, Mulay, & Colman, 2018). The majority of the studies sourced focused on populations in high-income nations including Australia. Results showed that suicide rates are elevated above non-Indigenous rates in many Indigenous populations worldwide with the highest at 50.4 per 100,000 among Aboriginal and Torres Strait Islanders in the Northern Territory of Australia.

In Australia, national data shows Aboriginal students have lower retention rates in school in both years 11 and 12 (the final years of schooling) with the year 12 retention rate being 55.1% compared to other students (82.9%) (AIHW, 2015). Employment, a protective factor against harmful drug and alcohol use (Spooner & Hetherington, 2005), is lower among Aboriginal people compared to other Australians (ABS, 2014), and lower incomes resulting from lower levels of educational attainment and employment are associated with higher rates of morbidity and mortality, including from drug and alcohol use (AIHW, 2015; Galea, Nandi, & Vlahov, 2004).

National health frameworks (Australian Department of Health, 2013; CoA, 2017), service delivery reviews (Haswell, Blignault, Fitzpatrick, & Jackson Pulver, 2013) and other research (Bennett, Green, Gilbert, & Bessarab, 2013; Gray, Saggers, Atkinson, & Wilkes, 2008; Laliberté, Haswell-Elkins, & Reilly, 2009; Nagel, Robinson, Condon, & Trauer, 2009; Phillips, 2003) highlight the need for Aboriginal and Torres Strait Islander-led holistic care that address multiple individual as well as family needs, strengthens cultural identity, and develops the capacity of the workforce and systems to address inequity. ‘Mainstream’ drug and alcohol treatment services are generally under-accessed by Aboriginal and Torres Strait Islander people (AIHW, 2011b) and evidence for how well they deliver ‘best practice’ for Aboriginal and Torres Strait Islander people is consequently missing from the peer-reviewed literature (Gray et al., 2014; Rowan et al., 2014; Taylor, Thompson, & Davis, 2010).

Internationally there have been very few robust outcome studies focussed on residential treatment outcomes for young people with drug and alcohol issues (Nathan et al., 2016; Nathan, Bethmont, Rawstorne, Ferry, & Hayen, 2016). Importantly, length of stay and program completion in residential programs has been found to be associated with improved outcomes in the short term with both adults and young people (Darke, Campbell, & Popple, 2012; Edelen, Slaughter, McCaffrey, Becker, & Morral, 2010; Galaif, Hser, Grella, & Joshi, 2001; Mills, Pepler, & Cribbie, 2013; Orlando, Chan, & Morral, 2003). However, the lack of comparison groups in many studies has been problematic for attributing causality (Muck et al., 2001; Tripodi, 2009; Williams & Chang, 2000). These challenges in study design are compounded in research with Aboriginal and Torres Strait islander people given their small numbers as a proportion of the general population (AIHW, 2019). This translates to small numbers accessing and being represented in the treatment population even though Aboriginal and Torres Strait islander people are over-represented in the population receiving drug and alcohol treatment with around 1 in 6 (16%) clients identifying as Aboriginal or Torres Strait Islander in Australian treatment data (AIHW, 2019).

Study designs with a comparison group and randomisation remain ethically and logistically challenging in drug and alcohol studies, especially with young people and furthermore with those who identify as Aboriginal or Torres Strait Islander (Muck et al., 2001; Tripodi, 2009; Williams & Chang, 2000). There has consequently been very few RCT studies of residential (including Therapeutic Community) treatment programs in the mental health field more broadly (Pearce et al., 2017), and none identified with young people in the drug and alcohol treatment literature. There are also no studies we have been able to identify internationally with a focus on Indigenous young people and their experience and outcomes following residential drug and alcohol treatment.

Studies of adolescents in residential drug and alcohol treatment centres in the United States have shown significant reductions in drug and alcohol use and crime, and improvements in social and psychological well-being post treatment, though findings are mixed and study designs variable (Battjes et al., 2004; Edelen et al., 2010; Hser et al., 2001; Muck et al., 2001; Williams & Chang, 2000). There have been few treatment outcome studies identified in Australia focussed on adolescents (Spooner, Mattick, & Noffs, 2001) and none specifically focussed on Aboriginal young people. With over half of the Aboriginal and Torres Strait Islander population being under 25 years of age (ABS, 2016), the need to identify effective treatment approaches for young Aboriginal and Torres Strait Islander people with problematic drug and alcohol use is urgent.

The current study focusses on a residential treatment program that aims to address young people’s individual needs in treatment and which includes program elements to connect Aboriginal young people to culture and community. This paper examines whether Aboriginal and Torres Strait Islander young people who stay in the program long enough to receive a potential benefit show improvement on key measures 3 months’ post-discharge from this program.

### The program

The residential treatment program is a therapeutic community (TC) approach modified for young people (aged 13–18 years) who have problems with drugs and alcohol (see Participants section). A TC approach utilises the live-in community as a treatment tool and catalyst for change, with emphasis on treating the whole person (De Leon, 2000). The program, which has a stay of up to 3 months, aims to create a supportive drug-free environment for young people (13–18 years old), to encourage them to develop skills to manage their lives and reduce their drug and alcohol use (Nathan, Rawstorne, et al., 2016).

The program takes a harm minimisation approach, not expecting all individuals to be abstinent post-treatment (AIHW, 2014). The goal is to ensure individuals establish a positive basis for life outside the programme, which may include improvements in mental health, stability in employment, accommodation, social and family life (Nathan, Bethmont, Rawstorne, Ferry & Hayen, 2016). The program uses individual and group therapy, vocational education and other TC elements to effect change (Nathan, Rawstorne, et al., 2016). This program also has a continuing care service post-discharge, for up to 3 years, which aims to support young people back in the community. The continuing care aims to help them maintain positive changes in drug and alcohol use, and mental health, and to find stable housing, employment and build positive connections and relationships in the community.

### Program participants

Young people are referred to the program from the juvenile justice system, and community (including self, family or case worker) with high levels of trauma, poor mental health, history or arrests and unstable housing (Dixson et al. 2018, Nathan, Bethmont, Rawstorne, Ferry & Hayen, 2016). Those admitted meet the Diagnostic and Statistical Manual of Mental Disorders, 4th Edition (DSM IV) criteria for substance abuse or dependence (American Psychiatric Association, 2013). DSM IV criteria rather than DSMV are used by the program to ensure consistency and continuity in the eligibility criteria and in data collection. Furthermore, while the DSM V provides clear criteria for ‘substance use disorder’ and severity indicators, it does not have the same distinction as the DSM IV between ‘substance abuse’ and ‘substance dependence’, which has been argued to not capture broader social dimensions of problematic drug use, particularly in adolescent populations (Falck, Nahhas, Li, & Carlson, 2012).

Young people are not eligible for admission to the program if they are not aged between 13 and 18 years, do not meet the DSMIV criteria for substance dependence or have a criminal justice history that means they are unlikely to be suitable for a residential treatment program with other young people. For example, a history of sexual assault convictions or multiple convictions for arson. Young people are also sometimes refused bail by the criminal justice system and are not able to be admitted to the program. Those who do not meet the DSMIV criteria for substance dependence are referred for outpatient counselling.

## Method

This analysis of Aboriginal and Torres Strait Islander young people aged 13–18 years admitted to the TC from 2007 to 2016 investigates personal characteristics, drug use patterns, and experiences such as arrests, self-harm and suicide attempts before admission to the program and 3 months’ post-discharge. Only those who stayed 30 days or more in the program were followed up at three-months post-discharge as the program routinely only follows up young people with a minimum stay of 30 days. This 30 day stay is considered by the program staff and management as a minimum time in treatment that is required to have any demonstrable impact. Additionally, this follow up rule enables the program to maximise the value of staff resources required for follow up balanced against expectations of client changes post treatment.

### Research questions

Improvements in the same domains as other published studies in the US may be expected, but given the lack of research among Aboriginal young people no hypotheses were posed.

Research questions:

1. Is there a significant reduction in drug and alcohol use 3 months’ post-discharge from the program compared to baseline?
2. Is there a significant improvement in psychological well-being including a reduction in self-harm and suicide attempts 3 months’ post-discharge from the program compared to baseline?
3. Is there a significant improvement in family and social functioning 3 months’ post-discharge from the program compared to baseline?
4. Is there a significant reduction in arrests for Aboriginal and/or Torres Strait Islander young people 3 months’ post-discharge from the program compared to baseline?
5. Is there a significant improvement in engagement in study or employment for Aboriginal and/or Torres Strait Islander young people 3 months’ post-discharge from the program compared to baseline?

### Ethics and governance

This study and the publication have been approved by the Aboriginal Health and Medical Research Council (AHMRC) Ethics Committee of NSW (Ref: 1144/15). Approval was also received from the Human Research Ethics Committee (HREC) of the University of New South Wales (UNSW) (Ref: HC13014). An Aboriginal Advisory Committee (AAC) guided the study, with representatives of Aboriginal organisations, researchers, staff and young people who have completed the program. All young people consented to their data being used in research.

### Study design and data collection

The study design was a pre/post treatment program follow up study. Data included baseline and three-month follow-up repeated measures for young Aboriginal and Torres Strait Islander people admitted to the program from beginning 2007 to end 2016 who stayed 30 days or more; this is the timeframe used to determine eligibility for 3 months’ post-discharge follow-up by the program. Data were collected over the telephone by program staff using an electronic database to enter responses to the assessment instrument.

### Measures

Drug use measures included current use (yes/no) of the three most common drugs: tobacco, cannabis and amphetamine type stimulants (ATS) (Nathan, Bethmont, Rawstorne, Ferry & Hayen, 2016), number of days drinking alcohol in the past month and number of drugs used. Other items included engagement in study or work, arrests in the previous 3 months, self-harm and suicide attempts in the last 3 months and time spent with family and with friends who do not use drugs. These measures have been described in further detail in a previous publication (Dixson et al., 2018). Nonsuicidal self-injury (NSSI) is defined as the deliberate, self-inflicted destruction of body tissue without suicidal intent which is also commonly referred to as self-harm (Zetterqvist, 2015). One dependence and two functioning scales were also a focus of analysis, described in more detail in Table 1. While these scores were developed for an adult population and not validated among Aboriginal and Torres Strait Islander populations, the program has used them for several years, with practice-wisdom suggesting they have been useful in measuring change.

**Table 1:** Scales included in the analysis

Scales	Constructs measured	Description of measurement scale
Severity of Dependence Scale (SDS)	Five-item questionnaire measuring psychological dependence on different illicit drugs. A score from zero to three is used for each item, and then totalled (Lawrinson, Copeland, & Indig, 2005). If a cut-off score greater than three is measured, this is an indication for problematic alcohol use (Lawrinson, Copeland, Gerber, & Gilmour, 2007). If a cut-off score greater than four is measured, this is an indication for problematic drug use (Martin, Copeland, Gates, & Gilmour, 2006; Topp & Mattick, 1997).	0 = never/almost never 1 = sometimes 2 = often 3 = always/nearly always The scores are added together. The higher the score, the more problematic drug use.
General Functioning Scale	A sub-scale of the Family Assessment Device (FAD), which consists of 12 items that measure the overall functioning and health of a family (Byles, Byrne, Boyle, & Offord, 1988; Epstein, Baldwin, & Bishop, 1983). A score is given to each item from one to four, depending on the individual’s response to a certain statement, which is then averaged. A total score of two or more is considered as problematic family functioning, where the higher the score, the more problematic the family’s overall functioning (Byles et al., 1988).	A 4-point Likert Scale is used, which includes 4 options: strongly agree, agree, disagree and strongly disagree. The responses for each measure are added and the total is divided by the number of items in each scale. The higher the score, the worse the levels of family functioning.
Social Functioning Scale (SFS)	Consists of six items measuring levels of social conflict and financial hardship, time spent with drug users and non-drug users and involvement in criminal activity (Lawrinson et al., 2005). A scoring from zero to three is provided and then summed. A higher score represents a greater degree of social dysfunction (Darke, Hall, Wodak, Heather, & Ward, 1992; Epstein et al., 1983; Lawrinson et al., 2005).	A scoring from zero to three is provided for each question, where all scores are summed. Some answers my not be applicable to some people so a coefficient based on non-applicable answers is multiplied by the sum-total (Darke et al., 1992; Epstein et al., 1983; Lawrinson et al., 2005).

### Data analysis

To measure statistical significance of dichotomous variables prior to admission and 3 months post-discharge, McNemar’s Test was used with *p <* 0.05 as the cut-off value for significance. Analyses using McNemar’s Test applied to: proportion of participants using ATS, cannabis and tobacco in the preceding month before admission compared with the month before follow-month; spending time with family and friends; self-harm and attempted suicide; engagement in work or study; and having been arrested in the preceding 3 months before admission compared with follow-up.

To measure statistical significance of ordinal variables, the Wilcoxon Signed Ranks Test was used with a cut off of *p* < 0.05 for significance. Analyses using Wilcoxon Signed Ranks Test applied to: frequency of alcohol and polydrug use; and scores from the Severity of Dependence Scale (SDS), Family Assessment Device (FAD) and Social Functioning Scale (SFS) with IBM SPSS Statistics Version 17 used to analyse the data.

## Results

From 2007 to 2016, 619 Aboriginal and Torres Strait Islander young people were admitted to the program; 247 stayed in the program for 30 days or more; 89 were successfully followed up 3 months post-discharge (36% response rate). Of the 89 followed up, 82 self-identified as Aboriginal (92.1%), three as Torres Strait Islander (3.3%) and four as both Aboriginal and Torres Strait Islander (4.6%).

### Description of study sample

As shown in Table 2, young males are significantly more likely than young females to come into the Programme for Adolescent Life Management (PALM) under a court order, chi-square χ^2^ (1) = 7.30, *p* = .007. There were no other differences between males and females. The table also shows males (36%) and females (50%) commonly reported living in three or more places in the past 6 months.

**Table 2:** Demographic and other key information about the study sample (*N* = 89)

	Male	Female	Total	P(Sig)
	59 (66.3%)	30 (33.7%)	89 (100.0%)	
	n(%)	n(%)	n(%)	
Engaged in employment or study before entering PALM	23 (62.2%)	14 (37.8%)	37 (100.0%)	0.487
Places lived in the past 6 months				0.108
1 or 2 places	38 (73.1%)	12 (30.0%)	52 (100.0%)	
3 or more places	21 (56.8%)	16 (43.2%)	37 (100.0%)	
Court involved	50 (74.6%)	17 (25.4%)	67 (100.0%)	0.0007
Days at Palm				0.504
31 to 60 days	28 (70.0%)	12 (30.0%)	40 (100.0%)	
More than 60 days	31 (63.3%)	18 (36.7%)	49 (100.0%)	
	M (SD)	M (SD)	M (SD)	
Age at start of PALM	16.68 (0.99)	16.46 (1.01)	16.63 (1.00)	0.147

### Comparison with adolescents lost to follow up

Given that only 89 of the original 247 (36.0%) Aboriginal and/or Torres Strait Islander adolescents who stayed in the program for 30 days or more were successfully followed up, it was considered important to compare these 89 people against the 158 (64.0%) who could not be followed up on key study variables. Of the original 247 people, there were 59 young women (23.9%) and 188 young men (76.1%), of which a higher proportion of young women were followed up (*n* = 30; 51%) compared with young men (*n* = 59; 31%), (Pearson chi-square = 7.38, df = 1, *p* = .007). There were otherwise no significant differences between those who were followed up (*n* = 89) and those who could not be followed up (*n* = 158) across key variables: arrests in the last 3 months (Pearson chi-square = 0.79, df = 1, *p* = .374), suicide attempt (Pearson chi-square = 1.32, df = 1, *p* = .25) or self-harmed in the last 3 months (Pearson chi-square = 0.05, df = 1, *p* = .831), spending time with family (Pearson chi-square = 0.83, df = 1, *p* = .361), time with friends who do not do drugs (Pearson chi-square = 2.83, df = 1, *p* = .093) and number of residences in the past 6 months (Pearson chi-square = 10.71, df = 5, *p* = .06). There was also no difference between the two groups in scores on the Severity of Dependence scale (t = 0.23, df = 1, 242, *p* = .822).

Among the population of Aboriginal and Torres Strait Islander young people who attended PALM for at least 30 days (*n* = 247), the number of days in residential treatment ranged from 20 to 120 days. The 89 participants in the study who were able to be followed up spent a significantly greater number of days in PALM (M = 66.01; SD = 21.05) compared with the 158 people who could not be followed up (M = 56.82; SD = 22.56, t (245) = − 3.15, *p* = .002).

### Main results

In the remaining set of analyses, baseline data (at commencement of PALM) are compared against follow-up data for the 89 Aboriginal and Torres Strait Islander adolescents who stayed in the program for 30 days or more and were successfully followed up.

### Drug use

The proportion of Aboriginal and Torres Strait Islander young people who reported use of ATS, cannabis and tobacco in the last month was compared prior to admission and 3 months’ post-discharge (Table 3). There was a significant change in the proportion of young people reporting the use of ATS ‘once or more’ in the month before admission compared with those reporting use in the previous month at follow up (exact McNemar’s test = 11.172, df = 1, *p* < .001). As shown in Table 3, 27% of young people reported using ATS prior to admission shifted to reporting ‘no’ use of ATS after attending the program – a positive shift. Even though there were 5.6% young people who reported using ATS at 3 months post discharge compared to prior to admission, there was a significantly greater proportion stopping than starting the use of ATS. Of the young people reporting no change in their ATS use – 57.3% reported ‘no’ use of ATS both prior to admission and 3 months after discharge from the program, while 10.1% reported using ATS ‘once or more’ both before and 3 months post-discharge.

**Table 3:** Treatment outcomes at three-month post-discharge (versus pre-admission)

**Used once or more in the last month**			
	Three months post-discharge		
Pre admission	Yes n (%)	No n (%)	Total n (%)
ATS			
Yes	9 (10.1%)	24 (27.0%)	33 (37.1%)
No	5 (5.6%)	51 (57.3%)	56 (62.9%)
Total	14 (15.7%)	75 (84.3%)	89 (100%)
Cannabis			
Yes	57 (64.0%)	22 (24.7%)	79 (88.8%)
No	4 (4.5%)	6 (6.7%)	10 (11.2%)
Total	61 (68.5%)	28 (31.5%)	89 (100%)
Tobacco			
Yes	60 (67.4%)	12 (13.5%)	72 (80.9%)
No	11 (12.4%)	6 (6.7%)	17 (19.1%)
Total	71 (79.8%)	18 (20.2%)	89 (100%)
**Spending time with family and with friends who do not use drugs**			
	Three months post discharge		
Pre admission	No/little time n (%)	Fair amount/ a lot of time n (%)	Total n (%)
Spending time with family			
No/little time	37 (41.6%)	14 (15.7%)	51 (57.3%)
Fair amount/ a lot of time	14 (15.7%)	24 (27.0%)	38 (42.7%)
Total	51 (57.3%)	38 (42.7%)	89 (100%)
Spending time with friends who do not use drugs			
No/little time	59 (66.3%)	19 (21.3%)	78 (87.6%)
Fair amount/ a lot of time	8 (9.0%)	3 (3.4%)	11 (12.4%)
Total	67 (75.3%)	22 (24.7%)	89 (100%)
**Attempted to end life or self-harmed**			
	Three months post discharge		
Pre admission	Yes n (%)	No n (%)	Total n (%)
Attempted to end life in the last three months)			
Yes	0 (0.0%)	18 (20.2%)	18 (20.2%)
No	3 (3.4%)	68 (76.4%)	71 (79.8%)
Total	3 (3.4%)	86 (96.6%)	89 (100%)
Self-harmed in the last three months			
Yes	4 (4.5%)	23 (25.8%)	27 (30.3%)
No	2 (2.2%)	60 (67.4%)	62 (69.7%)
Total	6 (6.7%)	83 (93.3%)	89 (100%)
**Currently engaged in work or study**			
	Three months post discharge		
Pre admission	Yes n (%)	No n (%)	Total n (%)
Yes	22 (24.7%)	15 (16.9%)	37 (41.6%)
No	16 (18.0%)	36 (40.4%)	52 (58.4%)
Total	38 (42.7%)	51 (57.3%)	89 (100%)
**Number of arrests in the last 3 months**			
	Three months post-discharge		
Pre admission	None n (%)	One or more n (%)	Total n (%)
None	16 (19.3%)	4 (4.8%)	20 (24.1%)
One or more	39 (47.0%)	24 (28.9%)	63 (75.9%)
Total	55 (66.3%)	28 (33.7%)	83 (100%)

Similarly, there was a significant difference in the proportion of young people reporting using cannabis ‘once or more’ in the month prior to admission compared with reporting at 3 months’ post-discharge (exact McNemar’s test =11.115, df = 1, *p* < .001) (Table 3). There was a significantly greater proportion stopping than starting the use of cannabis, following the program. About 25% of young people reported using cannabis before admission and subsequently reported ‘not’ using cannabis 3 months post-discharge. In comparison, 4.5% of young people reported not using cannabis in the month prior to admission and shifted to using cannabis at 3 months’ post-discharge. Most young people (64%) reported cannabis use both before and after the program.

There was no significant difference in the proportion of young people who stopped or started using tobacco (exact McNemar’s test =0.0435, df = 1, *p* = 1.00) (Table 3). Many of the young people (67.4%) reported using tobacco both before and after the program.

The frequency of alcohol and polydrug use prior to and at 3 months’ post-discharge was analysed and is presented in Table 4. There was a significant difference (Wilcoxon signed ranks test z = − 4.173, *p* < .001) in the number of days young people reported drinking alcohol in the last month (Table 4), with a decrease in the median days of alcohol use from 3 days reported 3 months’ post-program discharge compared to baseline, which had a median of 8 days. Between pre-and post-program measures, 52 young people reduced the number of days of alcohol use while 22 young people increased the number of days they used alcohol. Fifteen young people reported no change in the number of days of alcohol use between pre and post program.

**Table 4:** Changed scores on SDS, FAD and SFS scales and alcohol frequency and polydrug use prior to and at 3 months post discharge

	Pre-admission		Three months post discharge		Positive ranks	Negative ranks	Ties	Z	P
	Median	IQR	Median	IQR					
SDS	8.00 (*N* = 80)	4.00	4.50 (*N* = 80)	6.00	67	11	2	−6.050	0.000
FAD	2.50 (*N* = 57)	0.50	2.42 (*N* = 57)	0.54	31	23	3	−1.693	0.090
SFS	10.8 (*N* = 75)	6.60	10.5 (*N* = 75)	3.60	40	30	5	−1.493	0.136
Alcohol frequency	8.00 (*N* = 89)	19.00	3.00 (*N* = 89)	10.00	52	22	15	−4.173	0.000
Polydrug use	2.00 (*N* = 89)	2.00	2.00 (*N* = 89)	1.00	51	15	23	−4.985	0.000

There was a significant overall reduction (Wilcoxon signed ranks test z = − 4.985, *p* < .001) in the number of drugs used prior to admission and 3 months post-discharge, as shown in Table 4. Comparing the number of drugs used pre and post, 51 young people reported a reduction in the number of drugs used, while 15 reported an increase, and 23 young people reported using the same number of drugs before and 3 months after program discharge.

The Severity of Dependence Scale (SDS) median score prior to admission was 8.00 which significantly decreased to 4.50 at three-month follow-up (Wilcoxon signed ranks test z = − 6.050, *p* < .001). Among the young people, the median score decreased for 67, increased for 11 and stayed the same for 2 people (Table 4). However, the post-discharge median of 4.50 is still indicative of problematic alcohol and drug use.

### Social and family functioning

Change in scores on two scales prior to and at 3 months’ post-discharge from the program was analysed (Table 4). There was no significant reduction in the mean scores on the Family Assessment Device (FAD) from baseline to three-month follow-up (Wilcoxon signed ranks test z = − 1.693, *p =* .090) and no significant difference in the Social Functioning Scale (SFS) mean score (Wilcoxon signed ranks test z = − 1.493, *p* = .136) prior compared to post-discharge.

There were no significant changes in the reporting of time spent with family before admission and 3 months post-discharge (exact McNemar’s test =0.000, df = 1, *p* = 1.00) (Table 3). There was also no significant change in the number reporting time spent with friends who did not do drugs prior to admission and 3 months’ post-discharge, (exact McNemar’s test =3.704, df = 1, *p =* .052). The majority of young people (66.3%) spent no or little time with friends who did not use drugs at both baseline and at 3 months’ follow-up.

### Suicide and self-harm

There was a significant change between pre-and post-reported suicide attempts by Aboriginal and Torres Strait Islander young people, (exact McNemar’s test =30.422, df = 1, *p* < .001) in the direction of fewer people attempting suicide at follow-up compared with baseline (Table 3). All 18 (20.2% of the sample) young people who reported attempting suicide in the 3 months prior to admission, reported no suicide attempts during the 3 months post discharge. There was also a significant reduction in the number of Aboriginal and Torres Strait Islander young people who reporting self-harming in the previous 3 months compared with follow up (exact McNemar’s test =17.640, df = 1, *p* < .001). Twenty-three young people (out of the 27 who reported self-harming in the 3 months prior to admission) reported no self-harming in the 3 months’ post discharge.

### Employment/study and engagement in crime

Aboriginal and Torres Strait Islander young people’s engagement in work or study and arrests in the previous 3 months were compared pre-and post-program (Table 3). There was no significant change in engagement in work or study prior to admission to the program and 3 months post-discharge (exact McNemar’s test =0.000, df = 1, *p* = 1.00). However, there was a significant decrease in the proportion who reported being arrested prior to admission and 3 months post-discharge (exact McNemar’s test =26.884, df = 1, *p* < .001). There were 47% of young people who reported one or more arrests prior to admission, shifting to reporting no arrests 3 months post-discharge. This was a greater proportion than the 4.8% of young people who reported no arrests in the 3 months prior to the program and were arrested during the 3 months post discharge.

## Discussion

This study examined how Aboriginal and Torres Strait Islander young people, following 30 or more days in a ‘mainstream’ residential drug and alcohol treatment program were faring after treatment. The analysis compared data at admission to community follow-up at least 3 months post-discharge. To our knowledge, this is the first study to undertake treatment follow-up measures with this population.

This study demonstrates therapeutic communities are an intervention worthy of further examination in relation to preventing self-harm and suicide among Aboriginal and/or Torres Strait Islander people, whose rates are among the highest in the world (Dudgeon et al., 2016). This study found a significant decrease in self-harm post compared to prior to the program among those who were able to be followed up. Reported suicide attempts also significantly decreased. However, it is important to acknowledge that attempted suicide or self-harm may have been the catalyst for entering the program, which may create a reversion to the mean as suicide attempts and self-harm may reduce regardless of the program.

Significantly fewer Aboriginal and Torres Strait Islander young people reported using alcohol again since completing the program, with fewer days using alcohol for those who continued to drink. Number of drugs used, and frequency also significantly reduced, and the Severity of Dependence Scale scores significantly improved. These results suggest the program and its components have contributed to reducing drug and alcohol use for at least some of the Aboriginal and Torres Strait Islander young people who participated in the program. Despite a significant reduction in the number using cannabis post treatment compared to baseline, 64% of the sample were still using cannabis at follow up. The reasons for the lack of impact on the use of cannabis among these young people requires further research. The improvements in life circumstances and reductions in related problems surrounding alcohol and drug use, such as arrests were found. It is well-known that Aboriginal and Torres Strait Islander young people are over-represented in youth detention, with a majority in adult prisons having previous periods of incarceration (ABS, 2017; Fox et al., 2013; Whitesell et al., 2013). The specific role of this program in possibly contributing to a reduction in arrests requires further investigation. Research shows imprisonment is costly not only to governments, but also to prospects of genuine rehabilitation as it erodes individual and community health (Francis, Cheryl Lero, & Daniel, 2011).

There were no significant differences for Aboriginal and Torres Strait Islander young people spending time with friends who did not use drugs or spending a fair or a lot of time with family pre-to post program. A change to peer networks can be important post treatment given the potential influence peer groups can have on young people’s drug and alcohol use (Brown et al., 2008; Engels, 2003). The reason why this change in peer networks did not occur for many of the young people in this study is not able to be determined from the current study data, but it may be the case that it takes more time than 3 months for young people to make connections with other young people who don’t use drugs. This may also be more challenging for Aboriginal young people as friends who use drugs may include those with whom they have kinship connections (Bennett et al., 2013).

The finding that time spent with family, often seen as an important source of support (Tsey et al., 2010), did not change is important and not necessarily a negative finding. For some young people, their family of origin may be a source of stress, and time away from them whilst in a TC and in the first few months post treatment discharge may help reduce pressure on relationships and contribute to improving family dynamics in the longer term (King et al., 2009; Waldram, Herring & Young., 2006). For those who did spend time with family, no significant improvements were recorded using the Family Assessment Device (FAD) 3 months after program discharge compared to before admission. With mean scores remaining above the clinically significant threshold, family is clearly a potential stressor for many of the young people who were in the program, as evident in other studies with Indigenous peoples’ (King, Smith & Gracey., 2009; Waldram, Herring & Young., 2006). Further attention to understanding the impact of family relationships in treatment and after care are needed. This is particularly important for Aboriginal and Torres Strait Islanders among whom ‘family’ often includes extended networks, not only immediate blood relatives. Aboriginal and Torres Strait Islander people have particularly enduring and instrumental kinship connections vital for identity, belonging and cultural knowledge transfer (Bennett et al., 2013), which helps to counter frequent experiences of racism (Tsey et al., 2010) reduce alcohol and drug use and develop resilience and strength (Brady, 1995). The conceptualisation of family in the tool used in the current study may not capture the broader notion of family for many Aboriginal and/or Torres Strait Islander people.

The loss to follow-up in this study is a limitation. Loss to follow-up is an issue in many adolescent drug and alcohol treatment studies (Tripodi, 2009; Williams & Chang, 2000) and this is likely to be more challenging when these young people also identify as Indigenous. It is possible that the subgroup of program participants who were doing well post treatment compared to those who were not were more likely to respond to a follow-up survey request. However, even though the follow up group may not have been representative of all those who stayed 30 days or more, the findings that there was a significant reduction in key harms, including self-harm, suicide attempts, substance use and arrests is nonetheless an important contribution to the field. There is a complete lack of published data on Aboriginal and Torres Strait Islander young people following residential drug and alcohol treatment and the current study suggests there could be positive outcomes for these young people from such programs. However, based on the current study design, causality cannot be attributed to the program, and any improvements could also be due to time or some other factor not measured in the study.

A comparison was made between those who were followed up and those lost to follow up and no differences were found on key socio-demographic measures or among key variables which were the focus of the analysis except that a higher proportion of young women were followed up compared with young men. This is a limitation of the study. Young men are possibly harder to follow up in the community as at baseline young men were significantly more like to be court involved than young women and this difference may persist post treatment for those young men lost to follow up. Additionally, those lost to follow up had a significantly shorter length of stay than those who were followed up.

The current study was focussed on those who had a length of stay of 30 days or more given the relationship between length of stay and outcomes (Darke et al., 2012; Edelen et al., 2010; Galaif et al., 2001; Mills et al., 2013; Orlando et al., 2003). A further analysis of the factors associated with retention in programs for Aboriginal and Torres Strait Islander young people would be useful to inform program design to better meet client needs and improve length of stay.

A further limitation is that the measures used were not developed specifically for Aboriginal and Torres Strait Islander young people. The service has used the best available and accepted measures in the field and the psycho-social measures have been validated in a range of studies as detailed in Table 1. Further, the changes found pre/post in the current study were in response to direct questions asking about behaviours, such as drug use, arrests, and self-harm rather than psychological scales with multiple items. Where scales were measuring more complex constructs, such as family functioning, we found no change, and this may or may not reflect a problem with the scale itself and its applicability to Aboriginal and Torres Strait Islander young people as discussed above. The strengths of this study include the co-design and collaboration with Aboriginal community organisations.

### Implications and future directions

Findings of this study provide some support for the capacity of ‘mainstream’ drug and alcohol residential treatment programs to provide positive outcomes for Aboriginal and Torres Strait Islander young people. Culturally-relevant modes of treatment and support are particularly important when Aboriginal and Torres Strait Islander young people are over-represented in the client group compared to in the community population (Gray et al., 2014; Taylor et al., 2010) as is the case with this program. In addition, there is a need to formally review programs to understand how they incorporate culturally relevant modes of caregiving to improve outcomes among young Aboriginal and Torres Strait Islander people. There is also a need to develop a robust tool for the measurement of outcomes post drug and alcohol treatment specifically for young people, including domains of life improvement that may be particularly relevant for those who identify as Aboriginal and Torres Strait Islander.

## Conclusion

This study followed up Aboriginal and Torres Strait Islander young people after attending a residential alcohol and drug treatment program in Australia. Given the limited evidence about outcomes among these young people following treatment, this study makes and important contribution to the field. Several significant improvements were found in reduced drug use, arrests, self-harm and reported suicide attempts, although study limitations suggest caution in attributing changes directly to the program. Nonetheless, the study provides some evidence for the effectiveness of a residential treatment program using a Therapeutic Community approach for Aboriginal and Torres Strait Islander young people with problematic drug and alcohol use.

## Data Availability

This publication has used sensitive health information. Except in the form of conclusions drawn from the data, researchers do not have permission to disclose any data to any person other than those authorised for the research project.

## Abbreviations

ABS: Australian Bureau of Statistics
AIHW: Australian Institute of Health and Welfare
ATS: Amphetamine type stimulants
CoA: Council of Australian Governments
FAD: Family Assessment Device
MCDS: Ministerial Council on Drug Strategy
PALM: Program for Adolescent Life Management
SDS: Severity of Dependence Scale
SFS: Social Functioning Scale
TC: Therapeutic Community
